# Machine learning for enhancing the ablation outcomes using recurrence quantification analysis in human persistent atrial fibrillation

**DOI:** 10.3389/fphys.2026.1831826

**Published:** 2026-09-16

**Authors:** Noor N. Qaqos, Fernando S. Schlindwein, G. André Ng, Xin Li

**Affiliations:** 1 School of Engineering, University of Leicester, Leicester, United Kingdom; 2 Information Technology Department, Duhok Polytechnic University, Duhok, Kurdistan, Iraq; 3 National Institute for Health Research, Leicester, United Kingdom; 4 Department of Cardiovascular Sciences, University of Leicester, Leicester, United Kingdom

**Keywords:** atrial fibrillation, catheter ablation, machine learning, mapping catheter, recurrence quantification analysis

## Abstract

**Background:**

Several ablation strategies, including dominant frequency (DF), complex fractionated atrial electrograms (CFAEs), and rotors, have been used to target the drivers of atrial fibrillation (AF). The success rate of these strategies remains suboptimal.

**Objective:**

This work aims to develop a model using recurrence quantification analysis (RQA) variables and machine learning (ML) algorithms to predict the responses of ablating intracardiac electrograms (EGMs) and their impact on terminating AF and cycle length changes.

**Methods:**

3206 non-contact EGMs were collected from 10 persistent AF patients. Two classes were considered as labels based on EGM’s responses to ablation (positive and negative responses). Nine RQA-based variables were extracted from the EGMs. Ten ML classifiers were trained and tested using leave one patient out a 10-fold cross-validation (LOPOCV)).

**Results:**

The decision tree outperformed other ML models, achieving a balanced accuracy of 73.46%, F1_score of 74.05, and AUROC of 0.74. The high performance was achieved using the three most important features (determinism, longest diagonal line, and recurrence rate) with the importance of 30%, 23%, and 13%, respectively. Statistical analysis showed high values of all median RQA variables for the EGM’s negative responses over the positive ones.

**Conclusions:**

Our results show that RQA variables can effectively highlight the electrophysiological differences between EGM positive and negative responses to catheter ablation. The model might aid cardiologists in predicting ablation outcomes and reducing the number of unsuccessful ablations. A comparison between the proposed approach and previous works shows the superiority of this model.

## 1 Introduction

Atrial fibrillation (AF) is the most common cardiac arrhythmia, affecting about 1-2% of people around the world and increasing the risk of stroke up to 5-fold (Hindricks et al., 2021). Although ablation of AF substrate has become an established therapy option for AF patients, there are no available guidelines for standard protocol ablation strategies. Besides pulmonary vein isolation (PVI) (Haissaguerre et al., 1998; Hindricks et al., 2021), which is the cornerstone of ablation protocols for different types of AF, ablation protocol strategies for persistent and long-standing AF types are more complicated. So far, no unique approach has been uniformly proven in patients with persistent AF. Classical strategies have been used to quantify the intracardiac electrogram (EGM) signals to target AF drivers in the literature such as dominant frequency (DF) (Sanders et al., 2005), organization index (OI) (Takahashi et al., 2006), regularity index (RI) (Sanders et al., 2005), complex fractionated atrial EGMs (CFAEs) (Nademanee et al., 2004), focal impulse and rotor mapping (FIRM) (Zaman et al., 2014), and the entropy index. However, these methods are still far from satisfactory for persistent AF because of different mechanisms that are responsible for initiating and maintaining the AF, such as the focal ectopic (Haissaguerre et al., 1998), multiple wavelets hypothesis (Konings et al., 1994), single and multiple re-entry circuits (Jalife et al., 2002), and the rotors that initiate the functional re-entry (Narayan et al., 2012). Therefore, for ablation procedures, the current analysis tools might have failed to provide a definitive solution for persistent AF treatment because they have not been capable of fully describing the complexity of the AF dynamic behaviors.

Non-linear techniques have been applied in various fields, including biology and medicine, and are based on concepts of chaos (Webber, 1991). The dynamic behavior of cardiac signals for different types of arrhythmias has been widely investigated using recurrence analysis tools, particularly when considering the advantages of recurrence plots (RPs) and recurrence quantification analysis (RQA) variables for characterizing non-stationary time series data (Zbilut et al., 2002), and in general unveiling the dynamic underlying phenomena (Marwan et al., 2007, Marwan et al., 2002). The RQA properties outline as the singular framework for analyzing the cardiac signals in a broad sense over the traditional approaches due to their possibility to quantify the (i) signal regularity, determinism and laminarity, (ii) dynamic topological invariants, e.g., Kolmogorov-Sinai entropy (Faure and Korn, 1998), (iii) mutual information theory (Marwan et al., 2007; Soriano et al., 2011), and (iv) information theoretic measures such as generalized entropy (Prichard and Theiler, 1995).

Recurrence is one of the main characteristics for describing dynamic systems and was built by Poincaré in 1890. RPs have been applied to time series data to provide a visualization representation of the temporal domain of the signals. It can be used to visualize non-stationary signals, making it a convenient analysis tool for physiological data. These techniques have been widely applied to nonlinear medical data, and EGM signals have been shown to have dynamic behavior (Hoekstra et al., 1995). RP analysis later was found to be a promising approach to studying the temporal and spatial behavior of complex nonlinear systems (Spiridonov, 2017). Therefore, it has been used to study EGM behavior due to its spatiotemporal relation (Thanigaimani et al., 2017). Nonlinear techniques have provided new methods for analyzing and understanding the behavior of EGM signals. Several works utilized RQA variables for detecting cardiac arrhythmias such as AF. Moreover, the recurrence analysis tool explores the EGMs over a period of time, making it helpful in identifying the changes in signal characteristics. Hence, it has been widely used to analyze the spatiotemporal behavior of EGMs, which plays an important role in AF.

Within the frameworks of RP and RQA, several works have shown the use of RQA variables to analyze and characterize the cardiac atrial signals (Censi et al., 2000). Censi and colleagues have characterized recurrence patterns in two different atrial sites during chronic AF in 19 patients using non-contact EGM signals, indicating the theoretical paradigm and coupling in the atrial functions. Sun and Wang have developed a new algorithm to predict AF termination using nonlinear analysis, and their work provides a new perspective on an accurate method for predicting the spontaneous termination of AF (Sun and Wang, 2008). Nicolas and colleagues have developed a machine learning classification model for detecting CFAE sites and investigating the temporal dynamics of EGMs during AF (Navoret et al., 2013). Stef Zeemering and colleagues (Zeemering et al., 2015) have quantified and analyzed the spatiotemporal dynamical behavior of atrial activation sites in high-density AF human atrial recordings, suggesting that the RQA parameters were able to differentiate among three categories (persistent AF, paroxysmal AF, and no history of AF). More recently, Baher et al. have differentiated EGMs representing sites of active AF drivers from those of passive sites, suggesting a higher EGM conformation in active over passive sites (Baher et al., 2019). In the latter, Almeida and colleagues characterized the dynamic behaviors of atrial activations from bipolar EGM data collected by CARTO system in 18 patients with persistent AF (Almeida et al., 2018). They analyzed the EGM signals before and after PVI and roof line (RL) ablation, finding that (PVI+RL) affected the dynamics of the underlying atrial tissue, and the EGMs collected after the ablation procedure were more organized and less fractionated.

The nature of this problem ― catheter ablation outcomes have been suboptimal and unsuccessful ― suggests that recurrence analysis tools might be helpful in providing additional information about the underlying AF atrial substrate, characterizing and exploring the complex AF dynamic behavior, and potentially helping in the identification of persistent AF targets for ablation procedures. These require defining a consistent set of RQA features that take into consideration the specificities of EGM atrial signals and the theoretical methodology. The automated prediction tool for ablation outcomes based on RQA variables could help cardiologists during ablation procedures to avoid unnecessary ablations. To the best of our knowledge, there is no existing work that utilizes machine learning models to predict ablation outcomes using RQA variables derived from unipolar non-contact EGM signals. In this work, we aim to develop a model that quantifies changes in EGM signals using RQA variables, aided by machine learning classifiers for predicting ablation outcomes. A comparison was performed between the EGM positive and negative responses in the RQA variables using statistical analysis tools. Additionally, a comparison was made between the proposed method and other previous works to show the superiority of this method over others.

## 2 Materials and methods

The study was reviewed and approved by the local ethics committee at the University Hospitals of Leicester NHS Trust and local NHS research ethics committee. The patients/participants provided their written informed consent to participate in this study. This study was approved by the East Midlands - Leicester South Research Ethics Committee, approval number 13/EM/0227.

### 2.1 Data presentation and electrophysiological study

This study involved a dataset from 10 patients with persistent AF (male, aged 36-76). The patients were referred to Glenfield Hospital in Leicester for the first-time ablation, and all patients were in AF at the start of ablation procedure. Study approval was obtained from the ethics committee after obtaining full consent from all patients. The average ablation duration was 6.5 hours (range, 5–8 hours). Fifty-one locations of high dominant frequency (HDF) regions were identified and ablated before pulmonary vein isolation (PVI) procedure in all patients. A total of 3206 EGM signals were collected from these patients using a non-contact mapping catheter (Abbott, Ensite array, USA) from the left atrium (LA) for a time duration of 3 seconds (**Figure 1A**). Four out of ten patients had a free AF (3 flutter and one sinus rhythm). EGM data were collected pre- and post-ablation, then categorized into two classes: positive response to catheter ablation (AF termination or AF cycle length (AFCL) increased by ≥ 10ms), and negative response (AFCL increased by < 10ms). The patient characteristics recruited in this study are shown in the **Supplementary Materials** (**Supplementary Table 1**) (Chu et al., 2022).

**Figure 1:**
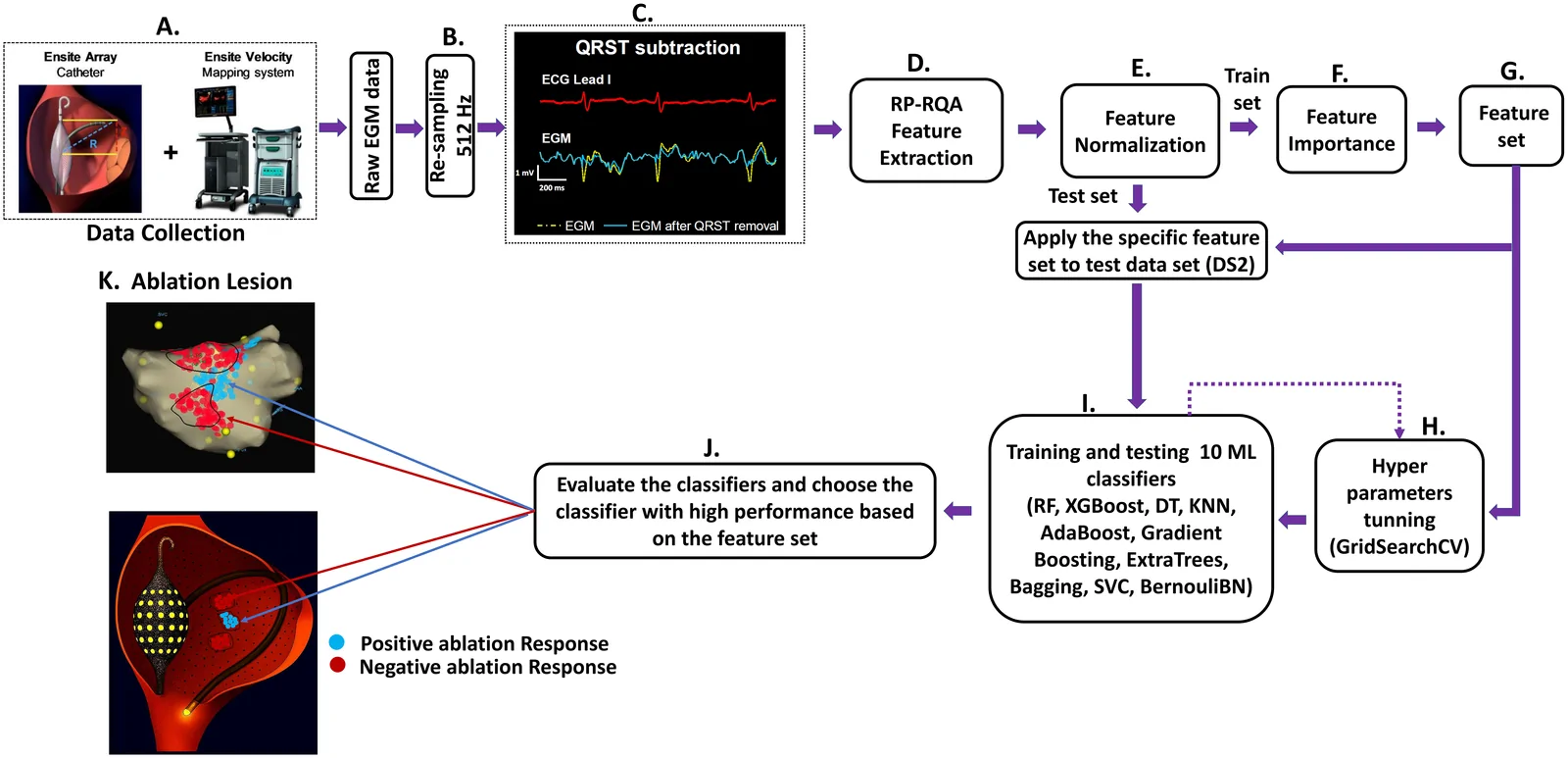
The pipeline of the proposed method. **(A)** Data collection using Ensite array catheter **(B)** Resampling the data to 512 Hz to reduce the processing time and also the memory allocation **(C)** QRST subtraction process. **(D)** Feature extraction using nine RQA variables. **(E)** Feature normalization using the standardization method. **(F)** Order the features based on their importance. **(G)** The feature sets that were used to train and test the model. **(H)** GridsearchCV technique for hyperparameters tuning **(I)** Training and testing the 10 ML models **(J)** Assessment to select the high-performance model. **(K)** Predicting the local atrial tissue responses to catheter ablation.

### 2.2 AF signal processing

**Figure 1** shows the pipeline of the proposed approach. The EGM signals were sampled at 2038.5 Hz and then resampled at 512 Hz using the cubic spline interpolation method to reduce the processing time and memory allocation (**Figure 1B**). A QRST subtraction process was performed to remove far-field effects resulting from ventricular activity (Salinet et al., 2013) (**Figure 1C**) (Li et al., 2018). This ventricular activity is not a part of the local atrial dynamics and can distort the EGM morphology, amplitude, and temporal structure, potentially affecting the RQA metrics. ECG lead I was used as a reference lead for the QRST cancellation process.

### 2.3 RP and RQA variables

RP is used to visualize the time-dependent behavior of the dynamic systems. It consists of a 2-dimensional matrix of 0 and 1 to visualize the recurrence state of a nonlinear system in the phase trajectory space.

RP aims to visualize the reappearance of states in the dynamic system based on the construction of a binary distance squared matrix associated with the value of ‘1’ when the distance is less than the threshold (*ε*) and ‘0’ otherwise. The black pixel is substituted for the ‘1’ values in the distance matrix.

Different complex patterns, such as diagonal, horizontal, and vertical dot patterns (Zbilut et al., 2002), can be seen from the RP matrix. Each of these patterns represents different mechanisms of a dynamic system. These patterns, which involve mixing and matching, give rise to different complexities, leading to the study of the quantification of the recurrence organization within the dynamic system. Therefore, RQA variables were established to quantify the system behavior based on the distribution of diagonal, vertical, and horizontal complex patterns with different lengths in the RP.

The Takens’ theorem can be used to construct the phase space trajectory for the time window of each EGM signal (Marwan et al., 2007), thereby revealing the hidden low-dimensional trajectory underlying the EGM signals. The axes for each signal will be delayed for the unidimensional EGM x(k), as illustrated in Equation 1:

(1) $$x(k)=[x(k),\;x(k-\tau ),\;\ldots ,\;x(k-(d_{e}-1)\tau )]$$

where: $d_{e}$= embedding dimension (the number of coordinates that resulted from the displacement of the time series x(k)).

τ = time delay of the time series for a certain number of times.

The set of these two parameters is crucial for studying the behavior of a dynamic system. The optimal τ and $d_{e}$ values were estimated in this work using the first local minimum points using a mutual information theorem, and the false nearest neighbor (FNN) test, respectively (Marwan et al., 2007). The 2-dimensional recurrence matrix ${\text{R}}_{\text{i},\text{j}}$ was defined based on the distance between the current and the delayed state up to the distance determined by the threshold (*ε*). Let us denote the state of nonlinear systems by $x_{i}$ at time i, then the square matrix $R_{i,j}$ for the N-length time series data can be formulated using Equation 2:

(2) $$R_{i,j}=\text{θ}\{\text{ϵ}-∥x_{i}-x_{j}∥\}$$

Where Θ {.} is the Heaviside function (the step function which gives 0 for the time ≤ 0 and 1 otherwise). *ε* is the cut-off distance (threshold parameter), which represents the most important parameter to build the RP. The ∥ ∥ is the norm, and the Euclidean norm was used in this study. Formula (2) can be written as Equation (3) (Coco et al., 2020):

(3) $$R_{i,j}=\{\begin{array}{l} 1:\;∥x_{i}-x_{j}∥\leq \epsilon \\ 0:\;∥x_{i}\;-\;x_{j}∥>\epsilon \end{array}\;\;i,j\;=\;1,2\;\ldots .,N$$

The binary patterns (‘1’ represented by black and ‘0’ by white) observed in recurrent plots characterize the dynamic behavior of the system. The diagonal lines are associated with the determinism of the system (high values represent more periodicity and more stability). In contrast, the vertical (or horizontal) lines reflect the laminar chaos of the system (more turbulence). For recurrence plot obtained from a time series data of length ( N), with a frequency distribution P(l)  of diagonal line length, frequency distribution P(v)  for vertical line length, probabilities p(l)  of finding the diagonal lines of length (l), probability p(v) of finding the vertical lines of length ( v), the RQA variables are associated with representative statistical measures of the recurrence matrix $R_{i,j}$ being illustrated as follows in Equations 4–12:

1. The density of the points in the RP, which represents the probability that the state recurs, referred to as recurrence rate (RR):

(4) $$RR=\frac{1}{N^{2}}\;\sum_{i,j=1}^{N}R_{i,j}$$

2. Determinism (DET), which represents the proportion between recurrence points that form diagonal structures (with length l ≥ $l_{min}$) in the RP:

(5) $$DET=\;\frac{\sum_{l=\;l_{min}}^{N}l\;P(l)}{\sum_{l=1}^{N}l\;P(l)}$$

3. Laminarity (LAM), which represents the proportion between recurrence points that form vertical structures (with length v ≥ $v_{min}$) in the RP:

(6) $$LAM=\;\frac{\sum_{v=v_{min}}^{N}v\;P(v)}{\sum_{v=1}^{N}v\;P(v)}$$

4. The average length of diagonal lines ( L) in the RP:

(7) $$L=\;\frac{\sum_{l=\;l_{min}}^{N}l\;P(l)}{\sum_{l=l_{min}}^{N}P(l)}$$

5. The length of longest diagonal line ( $L_{max}$):

(8) $$L_{max}=\text{max}(\{l_{i};i=1,2,\ldots ,\;N_{l}\})$$

6. The length of longest vertical line ( $LV_{max}$):

(9) $$LV_{max}=\text{max}(\{v_{i};i=1,2,\ldots ,\;N_{v}\})$$

7. The Shannon entropy of diagonal lines (D_ENTRO), which reflects the complexity of RP structure regarding the diagonal lines:

(10) $$D\_ENTRO=-\sum_{l=l_{min}}^{N}p(l)\text{ ln}p\;(l)$$

8. The Shannon entropy of vertical lines (V_ENTRO), which reflects the complexity of RP structure regarding the vertical lines:

(11) $$V\_ENTRO=\;-\sum_{v=v_{min}}^{N}p(v)\text{ ln}p(v)$$

9. The average length of the vertical lines, referred to as Trapping time ( TT):

(12) $$TT=\;\frac{\sum_{v=v_{min}}^{N}v\;P(v)}{\sum_{v=v_{min}}^{N}P(v)}$$

### 2.4 Optimal time-delay and embedding dimension

To determine the optimal time delay (τ) and embedding dimension ( $d_{e}$) parameters, different methods have been proposed (Cao, 1997). In this work, τ was calculated using the first minimum of mutual information, and the false nearest neighbor (FNN) test algorithm was used to calculate $d_{e}$. The FNN algorithm is based on the idea of increasing the dimension until the percentage of neighbors of the points stops decreasing in the reconstructed phase space trajectory (Zbilut and Webber, 1992). **Figures 2A, B** show the selection of optimal values for the time delay and the embedding dimension using mutual information and FNN algorithm, respectively. The optimal values of time delay and embedded dimension for reconstructing the RP structure for one of the real EGM signals used in this work are [0.0293 seconds, (15 samples)] and 3, respectively (**Figures 2A, B**). The state space trajectory plot is shown in **Figure 2C**.

**Figure 2:**
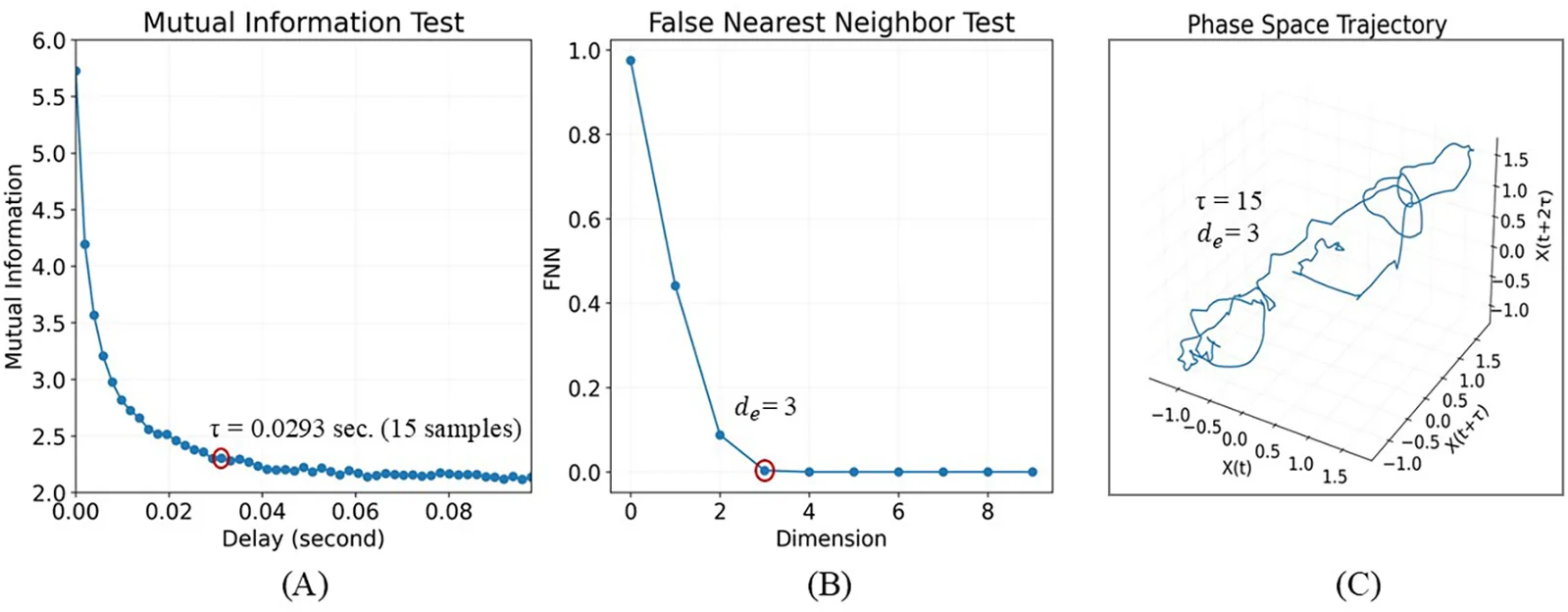
Selection the optimal values for **(A)** delay time, **(B)** embedded dimension, and **(C)** phase space trajectory plot for real EGM from patient 7 with positive response to catheter ablation.

### 2.5 Time-delay estimation

The time delay (τ) was determined from the first minimum of the average mutual information (AMI) curve for phase-space reconstruction. To evaluate whether the observed AMI structure could be associated with the finite-sample estimation bias, we generated 100 shuffled surrogate signals from EGM original signal and then recalculated the AMI curve. The curve of the original EGM remained substantially above the randomly shuffled surrogate distribution across the examined delays, suggesting persistent temporal dependence beyond the finite-sample bias floor. **Figure 3** shows the selection of time delay using AMI test.

**Figure 3:**
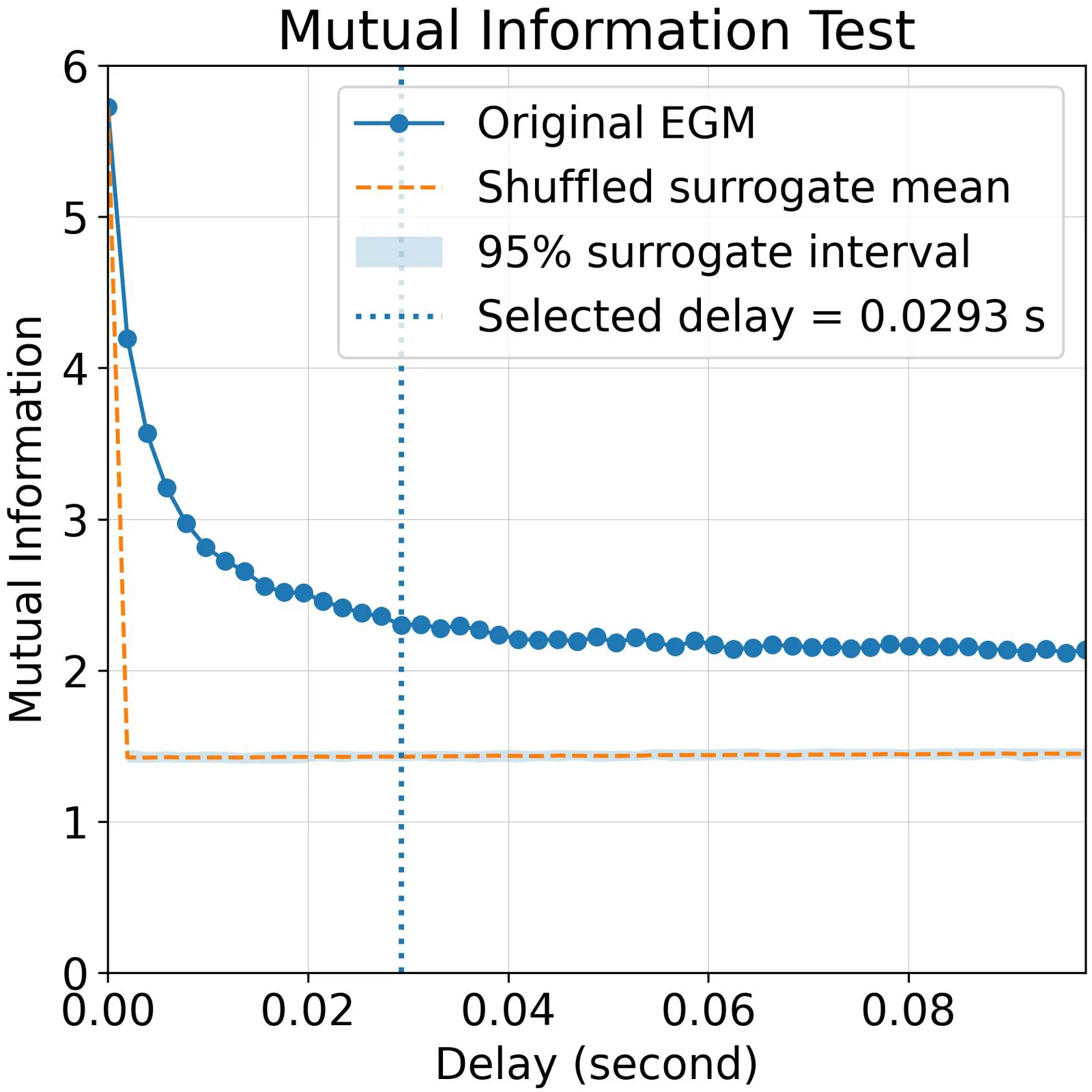
Selection of the time delay using the AMI. The solid line represents the AMI calculated from the original EGM signal. In contrast, the dashed line represents the mean AMI calculated from the 100 randomly generated surrogate signals generated from the original EGM. The shaded regions indicate the significance of 95% confidence intervals. The surrogate test preserves the amplitude distribution while disrupting the temporal dependencies to estimate the finite-sample bias floor.

### 2.6 Surrogate data analysis

To assess whether the EGM signals contain nonlinear deterministic structure that is not due to the noise, surrogate data testing was performed using the iterative amplitude adjusted Fourier transform (IAAFT). 100 surrogate signals were randomly generated for each EGMs, preserving the empirical amplitude distribution and power spectrum, while disrupting nonlinear temporal dependencies. The same parameters, including the time delay and embedded dimension used for the phase space reconstruction were applied to the original and surrogate signals, and the same RQA variables were applied to both. The RQA features obtained from the original EGM signals were compared with those obtained from surrogate signals. RQA variables were considered significantly different when the original value fell outside the 95% surrogate confidence interval. From the results shown in **Table 1**, the original EGM signals exhibit RQA characteristics that differed from their corresponding surrogate distribution. DET (0.93 vs. surrogate mean ± std = [0.86 ± 0.012]; z = [5.18]), LAM (0.97 vs. surrogate mean ± std = [0.95 ± 0.003]; z = [4.57]), D_ENTRO (2.19 vs. surrogate mean ± std = [1.86 ± 0.07]; z = [4.62]), L (6.22 vs. surrogate mean ± std = [4.54 ± 0.17]; z = [9.43]), LV_max (24 vs. surrogate mean ± std = [18.08 ± 4.13]; z = [1.43]), V_ENTRO (2.33 vs. surrogate mean ± std = [2.01 ± 0.08]; z = [3.99]), TT (5.42 vs. surrogate mean ± std = [4.37 ± 0.22]; z = [4.72], RR (0.005 vs. surrogate mean ± std = [0.004 ± 0.0003]; z = [1.83]) showed values for the original signals higher than their respective surrogate distributions. In contrast, L_max (163 vs. surrogate mean ± std = [132 ± 51]; z = [0.59]) remained within the expected range of their corresponding surrogate distributions. These findings indicate that the exhibited recurrence structure in EGM original signals, particularly DET and LAM organization, deviates from that of surrogates based on linear properties, providing evidence of nonlinear temporal organization of EGM signals.

**Table 1:** The RQA variables for the original EGM signals and their corresponding surrogate-generated signals.

Variable	Original	Surrogate mean	Surrogate STD	Z score	Empirical p value
RR	0.005161	0.004502	0.00036	1.833544	0.079208
DET	0.932941	0.869707	0.012201	5.182693	0.009901
LAM	0.970015	0.951912	0.003953	4.579979	0.009901
L	6.227749	4.54932	0.177848	9.437412	0.009901
L_max	163	132.49	51.42072	0.593341	0.514851
D_ENTRO	2.190178	1.863883	0.070617	4.620648	0.009901
TT	5.422636	4.373478	0.222089	4.724041	0.009901
LV_max	24	18.08	4.130889	1.433106	0.118812
V_ENTRO	2.335495	2.012192	0.081003	3.991258	0.009901

### 2.7 Optimal threshold (ε) parameter

Threshold (*ε*) is one of the most significant parameters to reconstruct the RP and RQA variables. If *ε* is too small, then only points in the RP that are close to each other will be considered as recurrence points. If *ε* is too large, then almost every point in the RP is the neighbor of every other point, even if they are not close to each other in time series data. Several methods have been used to find the optimal value for *ε*. Previous work by Schinkel et al., has suggested that *ε* should not exceed 10% of the phase space trajectory diameter (Schinkel et al., 2008). In this study, various threshold values were tested using the area under the receiver operating characteristic curve (AUROC) on the training dataset, and the recurrence threshold with the highest AUC from the ML models was selected to reconstruct the RQA variables. From **Table 2**, a threshold of 10% was selected as the optimal value for reconstructing the RP and calculating RQA variables.

**Table 2:** Selecting the optimal value for the threshold (ε).

Thresholds (%)	1	2	3	4	5	6	7	8	9	10	11	12
AUROC	0.52	0.57	0.60	0.60	0.61	0.69	0.67	0.67	0.70	**0.74**	0.68	0.67

The bold value represents the best AUROC result.

### 2.8 Feature extraction

A total of nine RQA variables (*RR, DET, LAM, L, L_max, LV_max, D_ENTR, V_ENTR, and TT*) were extracted using the pyRQA library that is embedded in the Python environment (Rawald et al., 2017) (**Figure 1D**). The optimal parameters were set using the methods explained in Sections 2.4 and 2.5. These parameters were used to quantify the EGM signals. In this work, the default values were used to define the minimum diagonal, vertical, and white vertical line lengths ( $l_{min}=\;v_{min}=\;v_{white}=\;$2), and the Euclidean distance was used as the similarity measure. A white vertical line length $(v_{white})\;$represents the number of consecutive time steps during which the system’s state does not repeat in phase space trajectory.

### 2.9 Feature normalization

Feature scaling is the pivotal pre-processing step in building the machine learning models. It is used to adjust the values of features to a specific scale. The normalization process is a significant step in ML because most models are sensitive to different feature scales. Therefore, standardizing the RQA features can improve both the performance and convergence speed of ML algorithms. In this work, standardization scaling transform (z-score) was used to normalize the RQA features (**Figure 1E**). The RQA variables are transformed to have a mean of 0 and a standard deviation of 1. Equation 13 was used to normalize the RQA variables:

(13) $$X^{\prime }=\;\frac{X-\;\mu }{\sigma }$$

Where: $X^{\prime }$ is the scaled feature value, X is the original feature value, μ and σ stand for the mean and the standard deviation of the feature, respectively.

The feature normalization process was performed using a pipeline implemented in Python (via the pipeline from scikit-learn). The scalar was fitted on the training data and subsequently applied to the test data to ensure that no information from the test set was used during training, thereby preventing data leakage. The normalization process was applied to each fold of the 10-fold dataset during both training and testing of the model.

### 2.10 Feature selection and importance

The RQA features from the train data set were sequenced and ordered using the embedded feature importance method (**Figure 1F**). The embedded selection feature approach in scikit-learn selects features based on importance scores learned from the trained model (the SelectFromModel method). For example, decision trees select features based on their ability to effectively partition the data into more homogeneous subsets. Features that produce the most informative and pure splits are considered more important. The feature importance was applied to the training data for each of the 10 folds. Mean Decreased Impurity (MDI) (often referred to as Gini importance) was applied to calculate feature importance. MDI counts the times a feature is used to split a node, weighted by the number of samples it splits (Perrier, 2015). The feature importance scores are normalized such that their sum equal one as in Equation 14:

(14) $$\sum_{i=1}^{f}{\text{feature importance}}_{i}=1\;$$

Where f represents the number of features, and in this study f = 9.

**Figure 4** illustrates the top-ranking features for predicting ablation outcomes. It can be seen that determinism (DET) had the highest importance over the others. After ranking the features based on their importance and contribution to a high accuracy space (Huang et al., 2023), nine sets of feature (feature set 1, 2, 3, …, 9) were constructed and used to train and test the ML models. Feature set 1 (FS1) includes the single most important feature (DET), feature set 2 (FS2) includes the two most important features (DET and L_max), and so on, with feature set 9 (FS9) containing all the features. These feature sets (FS 1, 2, 3,…, 9) were individually applied to train and test the models to evaluate the model performance on each feature set separately.

**Figure 4:**
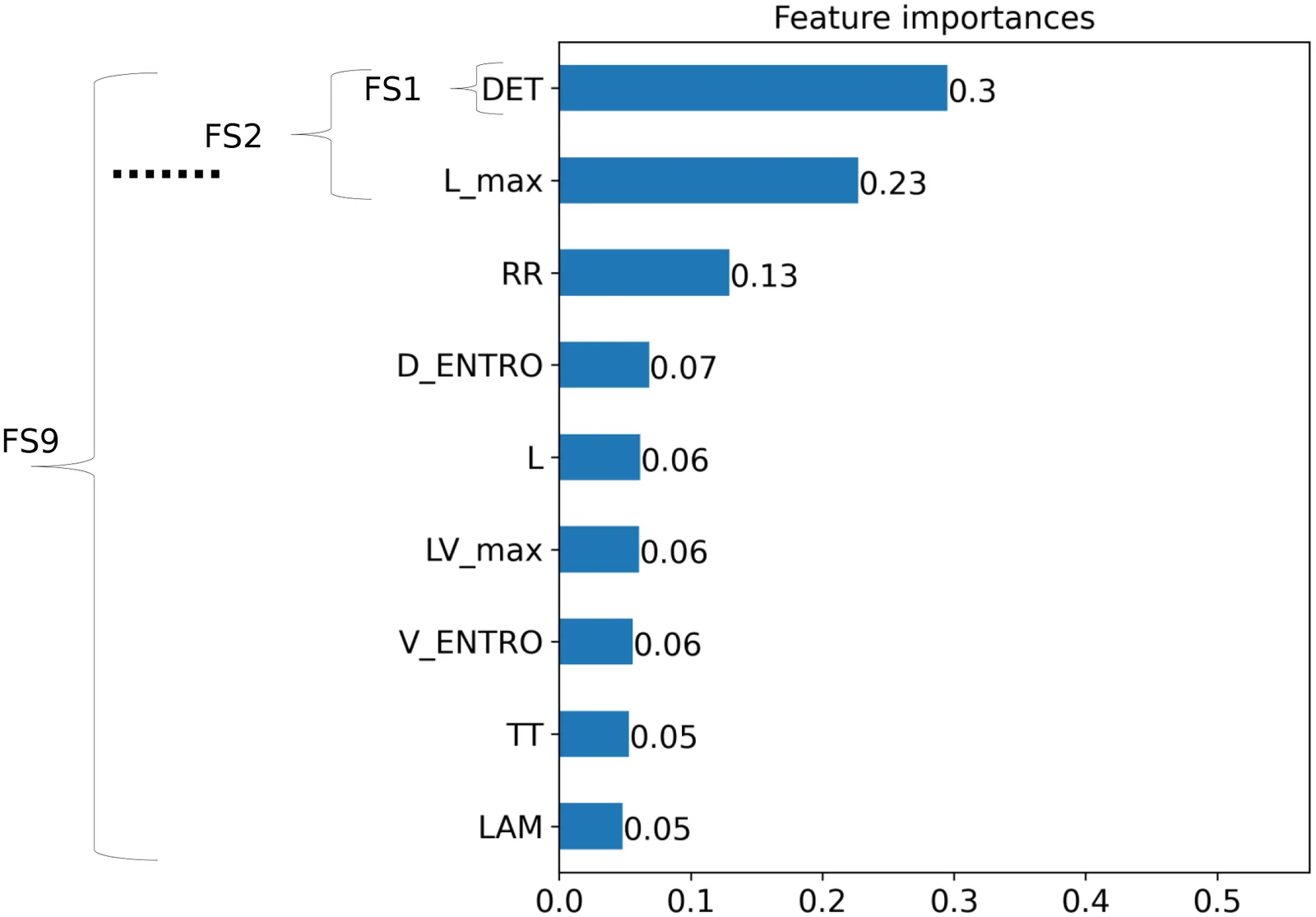
Top-ranking of the RQA features. FS1, FS2, …, FS9 are the feature set 1, 2, …9, respectively.

### 2.11 Model performance metrics

Several metrics, including (Overall accuracy, balanced accuracy, AUROC, sensitivity, specificity, precision, and F1_score), were used to assess the performance of the ten ML models. These metrics are illustrated as follows in Equations (15–21):

1. Overall accuracy is a measure of how often the model makes correct predictions, calculated as the total number of instances that are correctly identified divided by the total number of instances.

(15) $$\text{Overall accuracy}=\;\frac{\text{TP}+\text{TN}}{\text{TP}+\text{TN}+\text{FP}+\text{FN}}\times 100\%$$

2. Sensitivity (recall, true positive rate (TPR)) is a measure of the proportion of the positive instances that are identified correctly as positive.

(16) $$\text{Sensitivity }(\text{Recall},\text{ TPR})=\frac{\text{TP}}{\text{TP}+\text{FN}}\times 100\%$$

3. Specificity (true negative rate (TNR)) is the proportion of the actual negative instances that are identified correctly as negative.

(17) $$\text{Specificity }(\text{TNR})=\frac{\text{TN}}{\text{ TN}+\text{FP}}\times 100\%$$

4. Precision (positive predictive value (PPV)) measures the proportion of instances that are predicted as truly positive to all instances predicted as positive.

(18) $$\text{Precision}(\text{PPV})=\frac{\text{TP}}{\text{TP}+\text{FP}}\times 100\%$$

5. F1_score is an efficient metric to measure the performance of a model when imbalanced data are used, as it takes into account the type of errors FP and FN, which quantifies between the precision and recall values.

(19) $$\text{F}\_1_{\text{score}}=2\times \frac{\text{Precision}\times \text{Recall}}{\text{Precision}+\text{Recall}}\times 100\%$$

6. Balanced accuracy is a powerful metric for evaluating the model performance when the data are imbalanced, as it gives equal weight to the performance in all classes. It stands for the average of the sensitivity and specificity values.

(20) $$\text{Balanced accuracy}=\frac{\text{Sensitivity}+\text{Specificity }}{2}\times 100\%$$

7. Area under receiver operating characteristics curve (AUROC).

(21) $$\text{AUROC}=\int_{0}^{1}TPR(t)d(FPR(t)).\text{ and }FPR=\frac{FP\;}{FP+TN}$$

Where:

1. TP (True Positive): the positive instances that are correctly predicted positive.
2. TN (True Negative): the negative instances that are correctly predicted negative.
3. FP (False Positive): the negative instances that are incorrectly predicted positive.
4. FN (False Negative): the positive instances that are incorrectly predicted negative.

### 2.12 Supervised classification process

Ten ML classifiers were trained and tested for predicting the ablation outcomes. Gridsearchcv tool was used for optimizing and tuning the hyperparameters (**Figure 1H**). **Table 3** lists the hyperparameters, ranges, optimally selected values, and the performance evaluation based on the test data for the 10 defined ML classifiers. Other hyperparameters not shown in this table were at their default states. The RQA variables were assessed for normality using the Shapiro-Wilk test. After confirming the non-normality of the data variables, all RQA variables were expressed as the median and the interquartile distribution interval. The Mann-Whitney test was used to analyze the non-parametric unpaired RQA variables. The p-values smaller than 0.0001 were considered significant in the classification process.

**Table 3:** The hyperparameters and their optimal values.

ML models	Attributes	Ranges	Selected	Feature sets	Performance metrics
RF	N_estimators Criterion Max_depth	[‘entropy’, ‘gini’] [None, 3, 5, 7, 10] [100, 200, 300]	entropy 3 300	FS2	Accuracy = 72.24 Sensitivity = 80 Specificity = 65.50 Precision = 66.82 F1_ score = 72.82 AUROC = 0.76 Balanced accuracy = 72.75
XGBoost	N_estimators Learning_rate Max_depth	[50, 100, 200] [0.01, 0.05, 0.1] [None, 3, 5, 7, 10]	200 0.01 3	FS3	Accuracy = 71.18 Sensitivity=76.91 Specificity=66.20 Precision=66.40 F1_score=71.27 AUROC=0.72 Balanced accuracy = 71.56
DT	Criterion Splitter Max_depth min_samples_leaf min_samples_split class_weight	[‘gini’, ‘entropy’] [‘best’, ‘random’] [None, 3, 5, 7, 10] [50, 100, 150, 200] [2, 5, 10] [None, ‘balanced’]	‘gini’ ‘best’ 5 50 2 ‘balanced’	FS3	Accuracy = 72.74 Sensitivity = 83.69 Specificity = 63.23 Precision = 66.40 F1_ score = 74.05 AUROC=0.74 Balanced accuracy = 73.46
KNN	n_neighbors Weights Algorithm Leaf_size P	[3, 5, 7, 9, 11, 13, 15, 17, 19, 21] [‘uniform’, ‘distance’] [‘auto’, ‘ball_tree’, ‘kd_tree’, ‘brute’] [10, 20, 30, 40, 50, 60, 80, 100, 150, 200] [1, 2, 3]	15 ‘distance’ ‘auto’ 10 2	FS2	Accuracy = 67.94 Sensitivity = 69.93 Specificity = 66.20 Precision = 64.24 F1_ score = 66.97 AUROC=0.75 Balanced accuracy = 68.07
AdaBoost	N_estimators Learning_rate max_depth	[50, 100, 150] [0.01, 0.05, 0.1, 0.5, 1.0] [None, 3, 5, 7, 10]	50 0.01 3	FS2	Accuracy = 69.68 Sensitivity = 76.91 Specificity = 63.40 Precision = 64.60 F1_ score = 70.22 AUROC = 0.72 Balanced accuracy = 70.16
Gradient Boosting	N_estimators Learning_rate Max_depth	[100, 200, 300] [0.001, 0.01, 0.1] [None, 3, 5, 7, 10]	300 0.01 3	FS2	Accuracy = 69.87 Sensitivity = 76.38 Specificity = 64.22 Precision = 64.95 F1_ score = 70.20 AUROC = 0.73 Balanced accuracy = 70.30
ExtraTrees	n_estimators max_features max_depth	[100, 200, 300] [‘auto’, ‘sqrt’, ‘log2’, None] [None, 3, 5, 7, 10]	200 None 7	FS2	Accuracy = 67.84 Sensitivity = 75.91 Specificity = 60.84 Precision = 62.73 F1_ score = 68.69 AUROC = 0.76 Balanced accuracy = 68.37
SVC	C kernel degree gamma	[0.1, 1, 10] [‘linear’, ‘poly’, ‘rbf’, ‘sigmoid’] [2, 3, 4] [‘scale’, ‘auto’]	10 ‘rbf’ 2 ‘scale’	FS3	Accuracy = 69.28 Sensitivity = 77.79 Specificity = 61.89 Precision = 63.93 F1_ score = 70.18 AUROC = 0.74 Balanced accuracy = 69.84
Bagging	n_estimators max_samples max_features	[100, 200, 300] [0.5, 0.7, 1.0] [0.5, 0.7, 1.0]	300 0.5 0.7	FS4	Accuracy = 66.72 Sensitivity = 65.70 Specificity = 67.60 Precision = 63.78 F1_ score = 64.73 AUROC = 0.72 Balanced accuracy = 66.65
BernoulliBN	Alpha binarize fit_prior	[0.1, 0.5, 1.0, 2.0] [0.0, 0.5, 1.0] [True, False]	0.1 0.5 True	FS6	Accuracy = 64.85 Sensitivity = 79.66 Specificity = 51.98 Precision = 59.03 F1_ score = 67.81 AUROC = 0.58 Balanced accuracy = 65.82

RF, random forest; XGBoost, Extreme Gradient Boosting; DT, Decision Tree; KNN, K nearest Neighbor; SVC, support vector classifier; BernoulliBN, Bernoulli Naive Bayes.

## 3 Results

A total of 3206 EGMs were used to train and test 10 ML shallow classifiers. Nine RQA-based features were extracted and ordered based on their importance as measured by the contribution of each feature to the high-accuracy space. Ten-fold cross-validation was used in this work by taking into consideration the interpatient divisions (leave-one-patient-out cross-validation (LOPOCV). In other words, EGMs from the nine patients were used to train the models, and EGMs from 1 patient (remaining) were used to test it, to ensure the model is not biased. This process was repeated 10 times, and the performance of the models was calculated by taking an average of the metrics used (see **Figure 5**).

**Figure 5:**
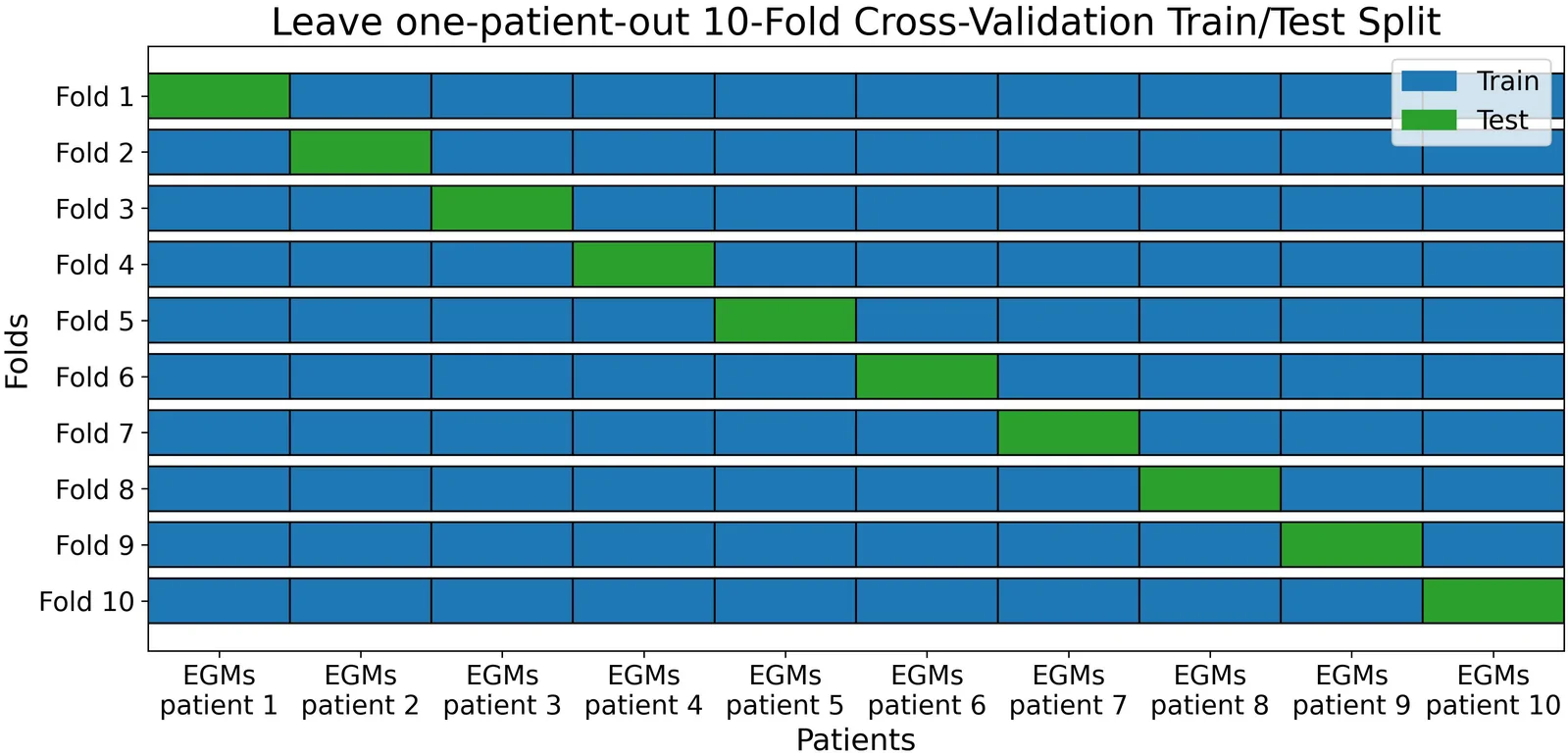
Splitting the data into train and test sets using leave EGMs one patient out a 10-fold cross validation (LOPOCV).

### 3.1 AF substrate characterization using RP

**Figures 6A, B** show the two EGM signals (positive and negative responses to catheter ablation) along with their respective distance matrices and RPs using the parameters mentioned above in Sections 2.4 and 2.5. These real EGM signals were collected from patient 7, who had an AF terminated. The difference between these signals can be found clearly. The RPs for the negative responses were characterized by the existence of a recurrence block and long diagonal lines, indicating high determinism in the negative response atrial signals. In contrast, the positive ones were characterized by a lower RR with residual diagonal lines.

**Figure 6:**
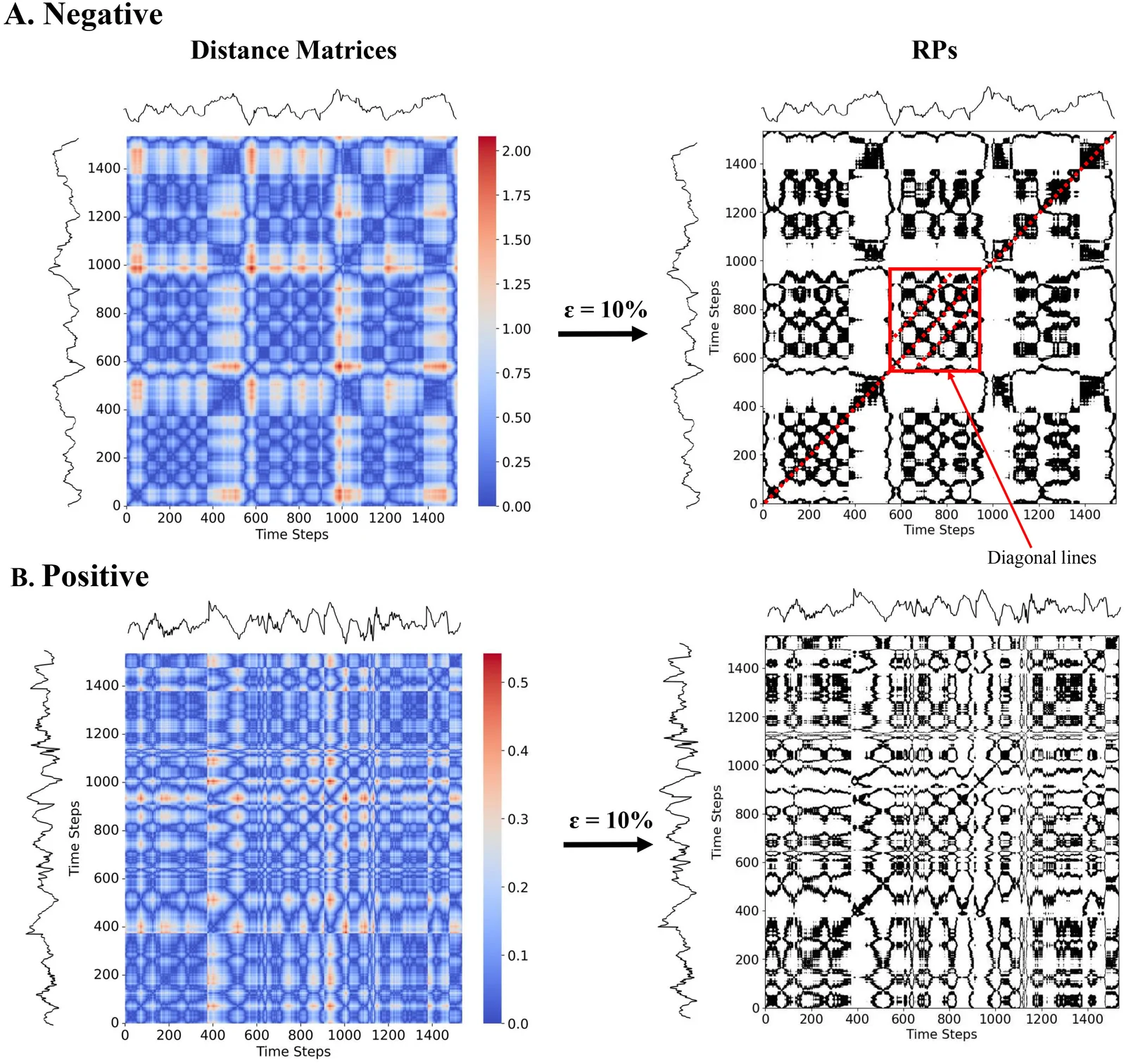
**(A)** and **(B)** are the distance matrices and the corresponding RPs for the real EGMs collected from patient 7 with the negative and positive responses to ablation, respectively.

### 3.2 EGM characterization using RQA variables

The effect of ablation outcomes (positive or negative) on the RQA-based variables is shown in **Figure 7**. All RQA variables were significantly affected in discriminating the outcomes (considering P-value <0.0001 for all variables). This indicates a significant difference in Electrophysiological (EP) characteristics of the atrial tissue, which yields positive results compared to adverse outcomes during the ablation procedure. In the context of the RR variable, as expected, it was higher in the sites of EGM negative responses (**Figure 7A**). The diagonal line of RQA variables (DET, L, L_max, D_ENTRO) was lower for EGM positive responses (**Figure 7B**). In the same context, the vertical line of LAM, LV_max, V_ENTRO, and TT was lower for the positive EGMs than the negative ones (**Figure 7C**). This indicates the presence of structures associated with deterministic behavior in positive ablation EGM signals. This is what was found in previous works by Tiago Almeida and colleagues, comparing and characterizing normal and fractionated EGMs (Almeida et al., 2018; Almeida et al., 2021), assuming normal as negative and fractionated as positive.

**Figure 7:**
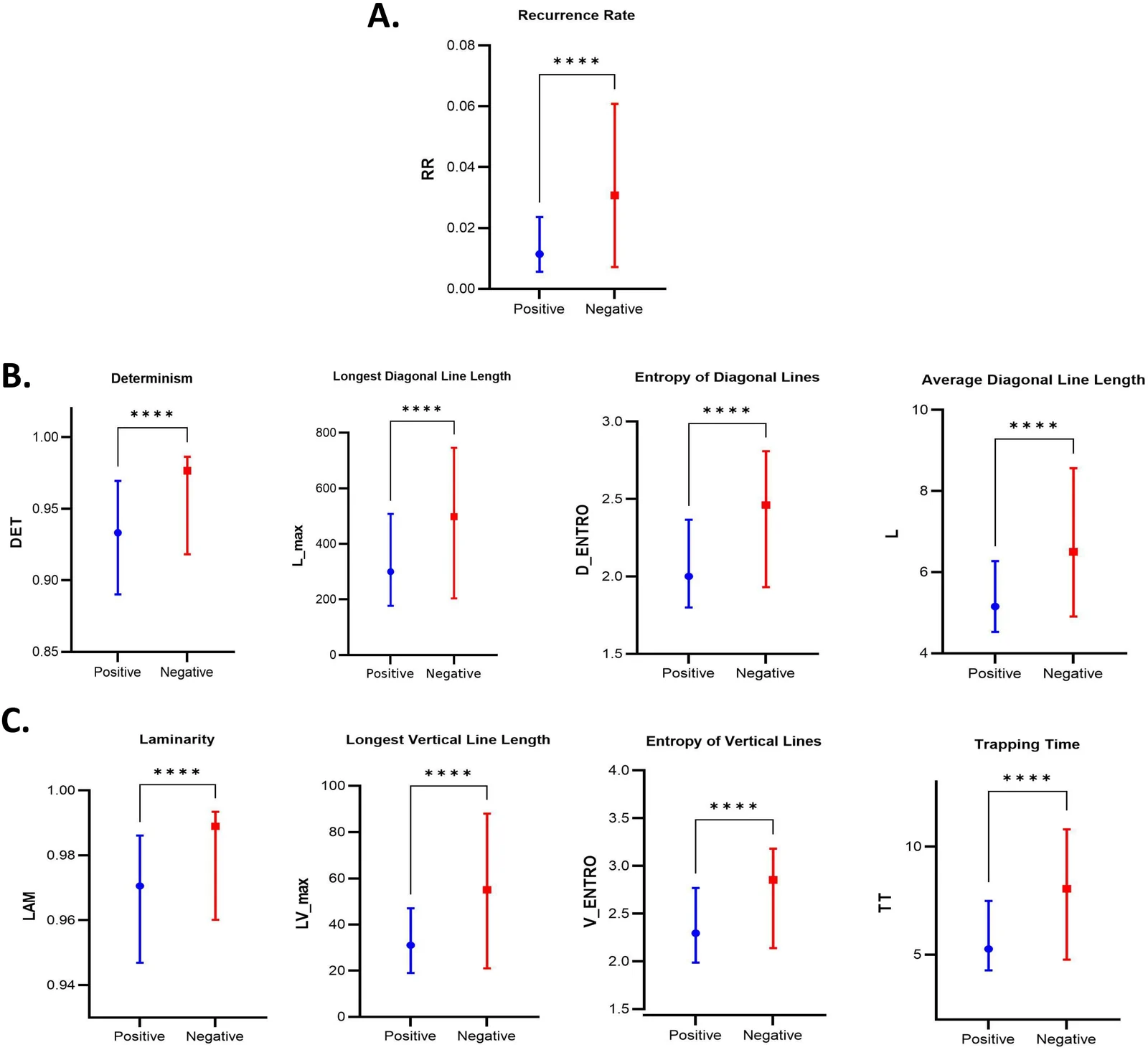
The RQA measures calculated from the EGM positive and negative responses to catheter ablation. **(A)** The RR variable, **(B)** and **(C)** are the variables related to diagonal and vertical lines, respectively. P, Positive; N, Negative.

### 3.3 Supervised classification process results

**Figure 8** illustrates the best performance of each model, achieved using the feature set (FS) mentioned in **Table 3**, as evaluated by the LOPOCV method. **Table 3** presents the seven metrics used to evaluate the models based on the optimized hyperparameters obtained using the gridsearchcv tool. Hyperparameter tuning was performed using GridSearchCV nested within a Pipeline to ensure that all preprocessing operations were executed within each cross-validation fold. By incorporating GridSearchCV into the pipeline, data transformations and model training were carried out separately for each of the ten folds, thereby preventing information leakage between training and testing data throughout the cross-validation process. This method ensures that feature selection, and model fitting are restricted to the training data in each fold during cross-validation. **Figure 9A** shows the classification accuracy for each patient, minimum, maximum, and the distribution of patient classification accuracies, while **Figure 9B** shows the mean and standard overall classification accuracy for all patients. As the non-contact EGM signals are contaminated by noise and the signal-to-noise ratio (SNR) can vary substantially between recording sites, this affects the selection of the time delay parameter. Therefore, we analyzed the classification performance across a range of time delays as shown in the **Supplementary Materials** (**Supplementary Figure 1**). The results show that the model’s performance remains stable over a reasonable range of time delays.

**Figure 8:**
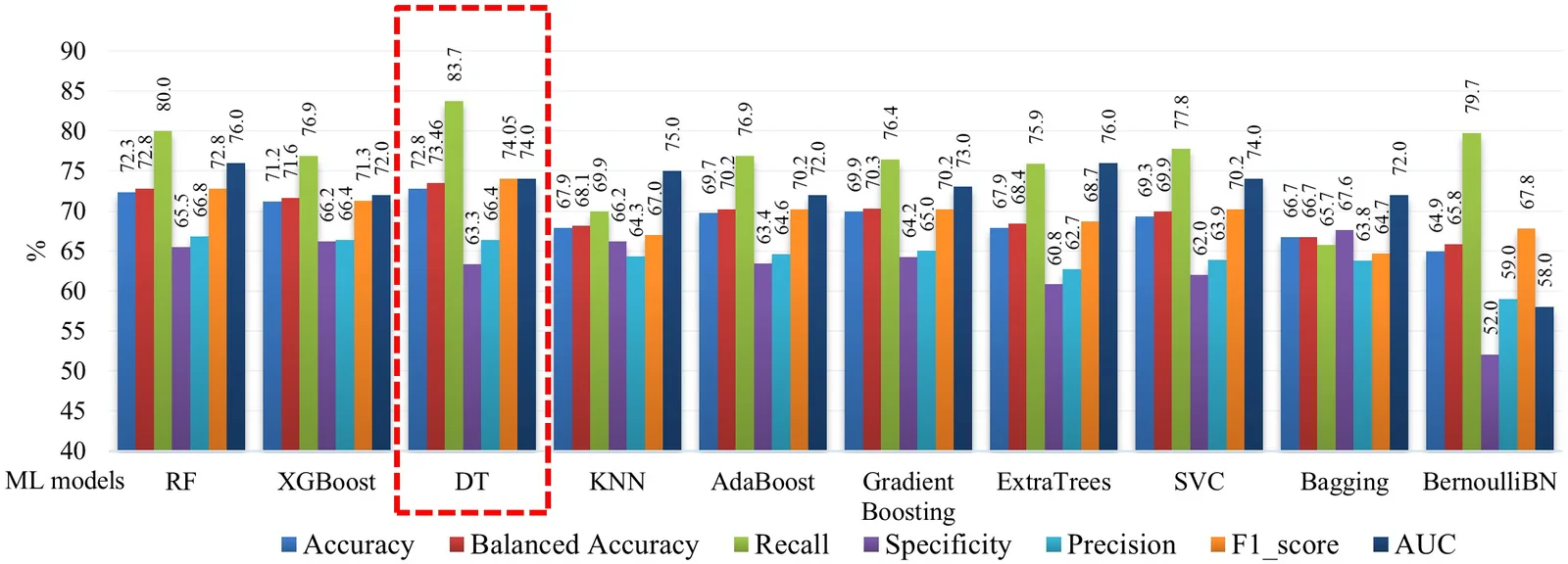
Models’ performance using test data set in the evaluating process.

**Figure 9:**
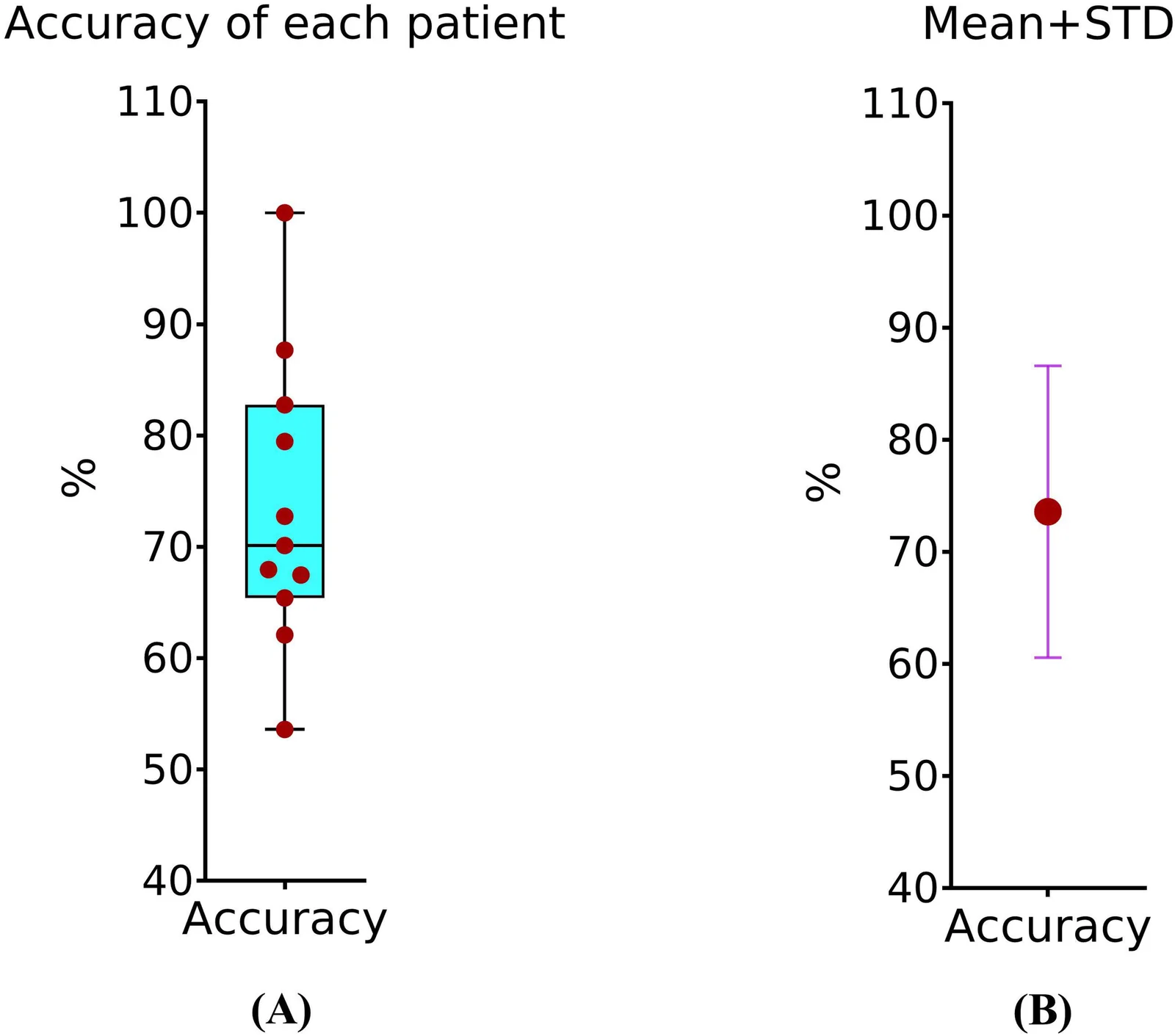
**(A)** the accuracy for each patient; **(B)** the mean and the standard deviation of the overall accuracy.

### 3.4 Highest-performing model

Three performance metrics (balanced accuracy, F1_score, and AUROC) were used to select the model with the highest performance (**Table 4**). The reasons for using thesis metrics to choose the highest-performing model are illustrated below:

**Table 4:** Evaluation metrics to select the highest-performing prediction model.

ML models	F1_score	Balanced accuracy	AUROC
RF	72.82	72.75	0.76
XGBoost	71.27	71.56	0.72
**DT**	**74.05**	**73.46**	**0.74**
KNN	66.97	68.07	0.75
AdaBoost	70.22	70.16	0.72
Gradient Boosting	70.20	70.30	0.73
Extra Trees	68.69	68.37	0.76
SVC	70.18	69.84	0.74
Bagging	64.73	66.65	0.72
BernoulliBN	67.81	65.82	0.58

The bold values represent the best-performing model based on three permanence metrics.

- The F1_score helps to make a balance between the recall and precision parameters.
- Balanced accuracy solves the imbalanced data problem by considering the performance of both minority and majority classes equally through considering sensitivity and specificity into account.
- AUROC balances the TPR and FPR, which means the model can correctly predict both the minority class (positive class (1490 EGMs)) and the majority class (negative class (1716 EGMs)), a pivotal for an imbalanced dataset. AUROC remains unaffected by the data imbalance due to its focus on how the model discriminates between the two classes rather than on how often it predicts the correct label. **Table 4** shows the performance of each model using these three metrics. It can be seen that the DT classifier outperformed other models, with the F1-score, AUROC, and the balanced accuracy were 74.05%, 0.74, and 73.5%, respectively.

### 3.5 CMs and the ROCs

The confusion matrix (CM) and the receiver operating characteristic (ROC) curve were used to assess the models. CM provides a comprehensive overview of the predicted values in comparison to the actual values. Also, it helps to visualize the errors, accuracy, and balance between TP and TN in the predicted values. **Figure 10** shows the CM for the highest-performing model (DT) using FS3 for training and testing the model. Additionally, the ROC is used to evaluate the performance of the models by plotting the true positive rate (TPR) against the false positive rate (FPR) using different threshold values (**Figure 11**).

**Figure 10:**
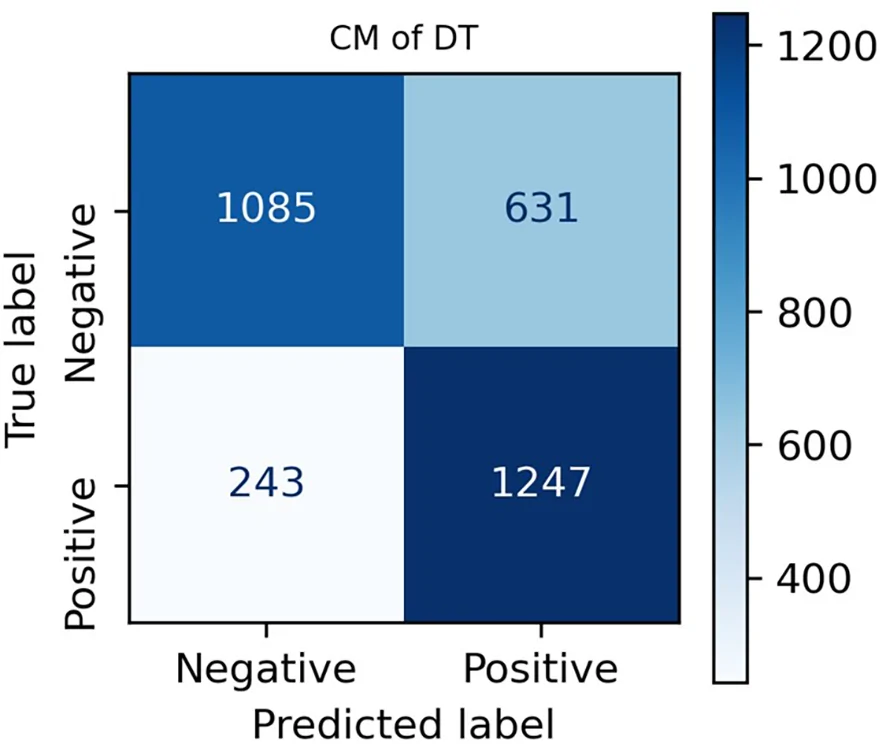
Confusion matrix for the DT model using FS3.

**Figure 11:**
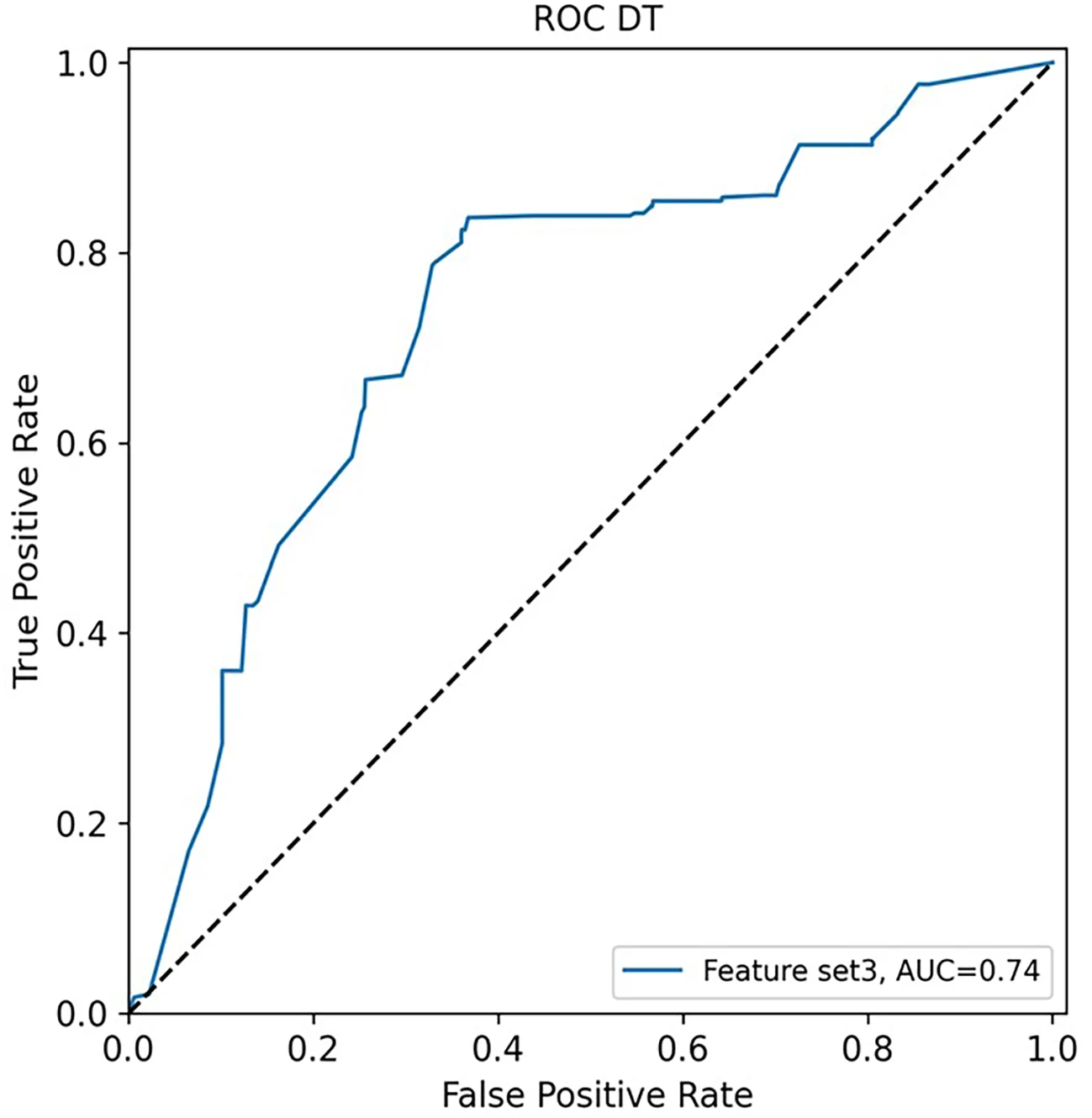
ROC and the AUC for the DT using FS3.

### 3.6 Model analytical representation

The best model was represented by analytical equations, which provided significant advantages in terms of interpretability, trust, and practical integration. The analytical models enable cardiologists to make decisions and personalize therapy based on both obvious and mathematically derived insights. Therefore, transience and clarity of the models can be the game-changers in improving patient ablation outcomes. The mathematical form of the developed DT model can be represented in Equation 22:

(22) $$\text{class}=\{\begin{array}{c} \begin{array}{c} 1\;\;:\;\;\text{if}(\text{DET}\leq 0.931\;\&\text{ L}\_\max>101\;\&\text{ RR}\leq 0.017)\\ 1\;\;:\text{ if}(\text{DET}>0.931\&\text{ RR}>0.009) \end{array}\\ \begin{array}{c} 1\;\;:\text{ if}(\text{DET}>0.978\;\&\;(481<\text{L}\_\max\;\leq 541)\;\&\text{ RR}>0.08)\\ \begin{array}{c} 0:\text{ otherwise} \end{array} \end{array} \end{array}$$

where class: 1=EGMs positive response.
class: 0=EGMs negative response.

The decision-making process and visualization of the DT model are presented in the **Supplementary Materials** (**Supplementary Figure 2**). The plot makes the model easy to understand and interpret how it works to predict or classify the data. Moreover, the tree structure fosters trust in the model’s predictions and also demystifies the black box nature of machine learning methods.

## 4 Discussion

The primary objective of the present study is to develop a tool (model) that aids cardiologists in predicting catheter ablation outcomes, thereby reducing the number of unsuccessful ablation attempts. The model was constructed using RQA variables, which have been employed previously to characterize and analyze the temporal behavior of biomedical signals. Our model was trained and tested using non-contact EGMs collected from 10 patients with persistent AF undergoing the ablation for the first time. Several points were identified, including the effectiveness of individual RQA variables in predicting ablation outcomes, the relationship between these variables in discriminating underlying changes in AF atrial activations, and an attempt to explore and investigate the persistent AF dynamic mechanisms.

Results from this study support recent findings concerning the dynamic behavior of AF arrhythmia and suggest that RQA-based variables can be used as an analysis tool to study AF dynamic behavior (Baher et al., 2019; Navoret et al., 2013; Almeida et al., 2018; Zeemering et al., 2015). In this study, nine RQA-based variables were extracted from the recurrence matrix and then used as the features to train and develop ML models. The main parameters were identified and optimized for the RQA variables to attempt to predict ablation outcomes in our patients. The results support the main findings from previous studies by Tiago et al., which suggest that RQA variables may be capable of discriminating between AF driver sites, represented by EGM fractionated signals, compared to normal ones (Almeida et al., 2018; Almeida et al., 2021). Our results are consistent with those reported by Almeida et al. (2018, Almeida et al., 2021), who demonstrated that RQA variables for normal EGMs (negative response) were higher than those for fractionated EGMs (positive response) using intracardiac contact mapping. Additionally, supporting recent findings that AF exhibits a dynamic behavior, and these variables may help in understanding the characteristics and dynamic mechanisms of AF (Zeemering et al., 2015; Baher et al., 2019).

### 4.1 RP-RQA for persistent AF EGM characterization

Several papers have utilized recurrence plots and RQA variables to study, analyze, and characterize the dynamic atrial behavior during AF (Zeemering et al., 2015; Baher et al., 2019; Navoret et al., 2013; Almeida et al., 2018). Our results represent a further step to (i) find the most important RQA variables for discriminating between EGM positive and negative responses records during the ablation procedure; (ii) assess changes in RQA variables for EGM positive and negative responses to ablation; (iii) select the optimal RQA parameters such as τ, $d_{e},\;$ and ε; and (iv) develop a model for predicting the ablation outcomes. Zeemering et al. have provided important new insights into the correlation between the AF Electrophysiology and the RQA-based variables for the existing wavelets in one AF (Zeemering et al., 2015). In our study, RQA variables were used to assess AF Electrophysiology using non-contact EGM signals that respond positively or negatively to catheter ablation in terms of AF termination and the change in AFCL. We observed significant differences in RQA variables when comparing the positive and negative ablation outcomes (**Figure 7**), indicating a meaningful relationship between the RQA attributes and the significant EP changes in the AF arrhythmia. Furthermore, the RR variable was higher for EGM negative responses to ablation in comparison with positive ones (**Figure 7**), which is an indication of more periodicity in the EGM-negative signal dynamics; or to put it differently, more organized electrical activity―negative responses―fewer harbors to AF drivers, and the organization index (OI) was significantly higher in the persistent AF patients who had successful AF termination. Similar findings have been reported as shown in several previous studies (Takahashi et al., 2006; Tuan et al., 2010). Moreover, the RQA attributes provided predictions of where to ablate to achieve positive ablation outcomes, thereby avoiding unsuccessful ablation procedures that can cause ongoing AF episodes and negatively impact patients in both their health and the treatment process. We believe it is essential to understand the changes in these variables and their relationship in the discrimination process. Therefore, this represents significant process for using the RQA variables to (i) characterize the EGM signals for ablation outcomes [e.g., AF termination (Sun and Wang, 2008)], and also (ii) study the changes of the RQA-based variables and their relation with the ablation outcomes.

The high residual deterministic components in the turbulent activations’ signals (positive EGM responses) could be due to unstable periodic orbits in the tumultuous motion (Marcotte and Grigoriev, 2017). The unstable sites may be represented by rotors that exhibit underlying dynamic behaviors during AF. Correspondingly, rotor sites are unstable in spatiotemporal domains, appearing and vanishing in different parts of the atria (Guillem et al., 2016).

### 4.2 EGM most important features

A quite comprehensive works have been reported from several previous studies regarding the use of RQA variables for discriminating and characterizing different types of arrhythmias using both invasive (Navoret et al., 2013; Almeida et al., 2018; Baher et al., 2019) and non-invasive mapping tools (Luongo et al., 2021). Indeed, the RQA variables have played a crucial role in the treatment of atrial fibrillation (Navoret et al., 2013; Baher et al., 2019). In our study, the highest-performing model (DT model) was obtained using only the three most important features, with DET being the most important feature. As expected, the positive EGM response provided a lower amount of determinism than the negative response signals. This finding indicates that recurrent patterns of these signals were observed less frequently and that they lasted for shorter time periods. These findings are consistent with Zeemering et al. (2015). They have reported that determinism in EGMs of persistent AF was lower than in those with no history of AF or paroxysmal AF (0.40 vs 0.59 and 0.62, p < 0.01) (Zeemering et al., 2015). In addition, this lower determinism is an indication of instability and non-periodicity in the EGM positive response signals. In contrast, the EGM negative responses to ablation exhibited higher determinism, indicating a highly organized region, with more stability, and periodicity. These markers are indicative of non-AF driver regions. Our findings also reflect observations reported by Tuan et al. (2010) that increased spectral organization plays a role in AF termination (Tuan et al., 2010). The second most important feature was (L_max). This feature was lower for the positive EGM responses to ablation, as expected, suggesting less periodicity and rapid fluctuations from the baseline, indicating higher-frequency content in these EGM signals (Atienza et al., 2009; Morillo et al., 1995). Our observations are consistent with the findings of Atienza et al. (2009). They observed that the ablation procedure significantly reduced the DFs in the left atrium, coronary sinus, and right atrium (Atienza et al., 2009). In summary, the number of neighborhood peaks was high for EGM positive response signals, indicating the rapid focal ectopic in these atrial activation regions (Qaqos et al., 2024b). This finding is consistent with our previous paper that suggest that neighborhood peaks of EGMs for negative responses were lower compared to positive responses (Qaqos et al., 2024b). The RR variable was higher for the negative than the positive ablation outcomes, indicating more recurrent patterns in the RP of EGM negative responses (represented by more black points) compared to the chaotic behavior of the positive response signals. The dense RP (high RR) suggests a high degree of determinism, reflecting regular dynamics in EGM negative responses. In contrast, the sparse RP indicates more chaotic or random behavior for the EGM positive responses (Marwan et al., 2007).

### 4.3 Comparisons with previous studies

A comparison has been made between our model and a number of previous studies in an attempt to demonstrate the effectiveness of our model in predicting ablation outcomes for persistent AF (**Table 5**). Although different datasets were used in previous studies, the purpose remains the same, allowing for meaningful comparison.

**Table 5:** Comparison between the proposed method and previous works.

Study (year)	Method used	Context/task	Labels	Signals used	AUC	Accuracy %	Sensitivity %	Specificity %	Precision %	F1_score %
Lankveld et al. (2016) ^45^	ECG frequency and time domain parameters + clinical parameters	Ablation outcomes at the level of patients	AF termination vs non-termination	ECG signals	0.74	N/A	N/A	N/A	N/A	N/A
Almeida et al. (2018) ^26^	RQA variables	RQA classification of AEGs (≥3.5 s signals, discriminating normal and fractionated EGM signals	Electrograms normal vs. Fractionated	Contact electrograms	Ranges between 0.70 and 0.72 depends on RQA variable used	N/A	0.679 - 0.716	0.359 – 0.390	N/A	N/A
Park et al. (2022) ^46^	fibrillatory wave amplitude (FWA) + Dominant frequency	Surface ECG (Dominant Frequency Derived from ECG Leads)	AF termination vs. non-termination	ECG signals	0.672	N/A	70.60	63.60	N/A	N/A
Feng et al. ^47^	shallow-wide ResNet architecture	Predicting ablation outcomes for the level of patient (not signals)	AF termination vs. non-termination	Contact electrograms	0.74	N/A	N/A	N/A	N/A	N/A
Qaqos et al. (2023a) ^48^	Spectral, temporal and statistical electrogram features + ML classifiers	EGM non-contact electrograms	Positive vs. negative response to ablation	Non-contact electrograms	0.71	72	64	77	61	67
**Proposed method (2025)**	**RQA variables + ML classifiers**	**EGM non-contact electrograms**	**Electrograms Positive vs. negative response to ablation**	**Non-contact electrograms**	**0.74**	**72.74**	**83.69**	**63.23**	**66.40**	**74.05**

The bold text represents the proposed method and its performance metrics.

### 4.4 Limitations and future studies

Results from this study represent a further step in developing a tool that can predict ablation outcomes as well as characterize RQA-based variables of non-contact intracardiac signals in terms of their responses to ablation. However, our study is limited by the type of data we have collected. Since we used non-contact EGM data, which were collected using the Ensite array balloon catheter (Abbott, USA), contact EGM signals should also be used to test and assess the performance of our model. Furthermore, our EGM signals were collected from a very small male patient population (only 10 patients), so analysis of a much larger dataset will be required to enrich our model and improve its performance. Our findings are restricted to 10 male patients and a specific ablation strategy that targets high-dominant frequency regions first, followed by the PVI procedure. Therefore, generalizing our findings to female patients or to other ablation strategies cannot be done directly. Further studies based on the contact intracardiac electrogram and the signals generated from biological computational models would also be helpful in validating and evaluating the proposed models in terms of ablation outcomes. The proposed method was based on analyzing the features from the temporal domain of the EGM signals. However, other spectral and statistical features should be considered to investigate the characteristics of these signals. Therefore, further studies should be conducted by incorporating additional features from our previous studies (Qaqos et al., 2024a, Qaqos et al., 2023a, Qaqos et al., 2023b) into these feature sets to assess any improvement in the models’ performance.

## 5 Conclusions

Here, we present a framework for developing and evaluating an analytical model to identify the electrograms that correspond to maintaining and perpetuating persistent AF, thereby representing potential targets for catheter ablation based on non-contact electroanatomic mapping approach. We have developed a model using RQA variables and ML tools to predict catheter ablation outcomes and have identified the most important RQA variables that are sensitive for discriminating between electrophysiology characteristics and predicting the EGM’s positive response from the negative counterparts. DT performance outperformed other ML models, achieving a balanced accuracy of 73.46%, an F1-score of 74.05, and an AUROC of 0.74. DET, L_max_, and RR were revealed to be the three most important variables for predicting positive and negative ablation outcomes. The nine RQA variables highlighted electrophysiological differences between the two post-ablation outcomes. Accordingly, we suggest that machine learning models, aided by RQA variables (DET, L_max_, and RR), could be used by cardiologists as a complementary tool to target AF drivers and predict catheter ablation outcomes during atrial substrate mapping procedures. This may assist cardiologists reduce the number of unsuccessful ablation points during the ablation procedure.

## Data Availability

The original contributions presented in the study are included in the article/**Supplementary Material**. Further inquiries can be directed to the corresponding author.
