# Correction: Machine learning for enhancing the ablation outcomes using recurrence quantification analysis in human persistent atrial fibrillation

**DOI:** 10.3389/fphys.2026.2009113

**Published:** 2026-10-02

**Authors:** Noor N. Qaqos, Fernando S. Schlindwein, G. André Ng, Xin Li

**Affiliations:** 1 School of Engineering, University of Leicester, Leicester, United Kingdom; 2 Information Technology Department, Duhok Polytechnic University, Duhok, Kurdistan, Iraq; 3 National Institute for Health Research, Leicester, United Kingdom; 4 Department of Cardiovascular Sciences, University of Leicester, Leicester, United Kingdom

**Keywords:** atrial fibrillation, catheter ablation, machine learning, mapping catheter, recurrence quantification analysis

In the published article, there was a mistake in the **Funding** statement. The correct **Funding** statement for this article is:

“The author(s) declared that financial support was received for this work and/or its publication. This work was supported by the NIHR Leicester Biomedical Research Centre, United Kingdom. GAN and XL is supported by British Heart Foundation Research Excellence Award (RE/24/130031), Medical Research Council Biomedical Catalyst Developmental Pathway Funding Scheme (MR/S037306/1) and NIHR i4i grant (NIHR204553). GAN is also supported by British Heart Foundation Programme Grant (RG/17/3/32774). Noor N. Qaqos received a PhD scholarship from the Higher Committee for Educational Development (HCED) in Iraq. This research did not receive any specific grant from funding agencies in the public, commercial, or not-for-profit sectors.”

The original article has been updated.

