# Supplementary material for: Machine learning for enhancing the ablation outcomes using recurrence quantification analysis in human persistent atrial fibrillation
Source: Front Physiol. 2026 Sep 16;17:1831826. doi: 10.3389/fphys.2026.1831826 (PMC13623697; doi:10.3389/fphys.2026.1831826)
Supplement: Supplementary file 1 [file SupplementaryFile1.doc]

Supplementary Material

# 1 Data presentation and Electrophysiological (EP) study

This study involved a dataset from 10 persAF patients (male, age ranged 36-76). The patients were referred to Glenfield Hospital in Leicester-UK for the first-time ablation procedure, and all patients were in AF at the start of the ablation procedure. Study approval was obtained from the ethics committee after obtaining the full consent from all patients. The ablation duration procedure was 6.5 (range 5-8) hours. A total of 3206 EGM signals were collected from these patients using a non-contact mapping catheter (Abbott, Ensite array, USA) from the left atrium. 51 locations of high dominant frequency (HDF) regions from all patients were identified and ablated before pulmonary vein isolation (PVI). Four out of ten patients had a free AF (3 flutter and 1 sinus rhythm). The EGM data were collected pre- and post-ablation, then labeled into two classes: positive response to catheter ablation (AF termination or AF cycle length ≥10ms), and negative response (AFCL increased <10ms). Table S1 shows the patients characterization and the AF within the period of 12 months following the ablation procedure (Chu et al., 2022).

# 2 Visualization of the tree plot

The DT plot is used to simplify the DT model by breaking down the decision-making process into a step-by-step and clear structure, where each split represents the decision rule for how the model works with the features, which makes it easy to understand and interpret how the model predicts or classify the data. Yes, on the right side of each node represents the true branch of condition (or threshold of the feature) and opposite to No on the left side of the nodes. Figure 1 shows the DT model plot. From this plot, we can see the most important features start from DET, followed by L_max, and finally the RR feature. The blue nodes (blocks) represent the EGM positive response, while the brown nodes represent the negative response (Géron, 2022).

# 3 ROC and the AUC

The ROC and the AUC for the 10 ML classifiers are shown in Figure 2. It can be seen the AUC for each feature set of data. The ROC curve is widely used to measure the binary classification model performance. A classifier represents good when its ROC curve bends towards the top-left. Therefore, its AUC will be high. The ROC is used when evaluating the classifier based on different threshold values. It also usually represents a good evaluation of imbalanced data (Géron, 2022).

# 4 Supplementary Figures and Tables

## 4.1 Supplementary Figures

**Supplementary Figure S1. Best performance of the model across a range of time delay**

Yes

No

**Supplementary Figure S2. The DT model visualization (the nodes with the blue colours represent the EGM positive response and with the brown colours the negative responses)**

**Supplementary Figure S3. The performance for each ML classifier vs the feature sets**

Supplementary Table S1. Patients’ characteristics and the AF recurrence within 12 months following ablation.

|  | **All patients** | **AF free at 12 months** | **AF-recurrence within 12 months** |
| --- | --- | --- | --- |
| **N** | 10 | 5 | 5 |
| **Age/years** | 57.7 ± 12.1 | 57.3 ± 9.0 | 58.2 ± 14.6 |
| **Body mass index/kg m^–2^** | 31.0 ± 5.7 | 32.6 ± 6.7 | 29.5 ± 3.7 |
| **Longstanding persistent AF** | 3 | 2 | 1 |
| **LA volume/ml** | 151 ± 38 | 146 ± 40 | 156 ± 35 |
| **Amiodarone usage** | 2 | 2 | 0 |
| **Hypertension** | 3 | 1 | 2 |
| **Diabetes mellitus** | 1 | 0 | 1 |
| **Previous myocardial infarction** | 1 | 1 | 0 |
| **Procedure duration/mins** | 389 ± 80 | 386 ± 65 | 393 ± 92 |
| **LA area ablated during HDF targeting/mm^2^ (% of LA total)** | 1362 ± 704 (7.3 ± 3.6) | 1055 ± 494 (5.9 ± 3.1) | 1670 ± 746 (8.7 ± 3.6) |
| **HDF occurrences ablated (%LA)** | 559 ± 268 (22.8 ± 8.7) | 448 ± 278 (21.6 ± 7.1) | 670 ± 205 (23.9 ± 9.9) |
| **Electrical cardioversion required at procedure end to restore sinus rhythm** | 5 | 0 | 5 |
| **Numbers are mean ± SD where relevant. HDF, highest dominant frequency; LA, left atrium.** | | | |

**References:**

CHU, G. S., LI, X., STAFFORD, P. J., VANHEUSDEN, F. J., SALINET, J. L., ALMEIDA, T. P., DASTAGIR, N., SANDILANDS, A. J., KIRCHHOF, P., SCHLINDWEIN, F. S. & NG, G. A. 2022. Simultaneous Whole-Chamber Non-contact Mapping of Highest Dominant Frequency Sites During Persistent Atrial Fibrillation: A Prospective Ablation Study. *Front Physiol,* 13**,** 826449.

GÉRON, A. 2022. *Hands-on machine learning with Scikit-Learn, Keras, and TensorFlow*, " O'Reilly Media, Inc.".
